# Application of an Event-Based Camera for Real-Time Velocity Resolved Kinetics

**DOI:** 10.1021/acs.jpca.2c00806

**Published:** 2022-03-23

**Authors:** Kai Golibrzuch, Sven Schwabe, Tianli Zhong, Kim Papendorf, Alec M. Wodtke

**Affiliations:** † Max-Planck-Institute for Multidisciplinary Sciences, Am Fassberg 11, D-37077 Goettingen, Germany; ‡ Institute for Physical Chemistry, Georg-August-University Goettingen, Tammannstrasse 6, D- 37077 Goettingen, Germany; § Institute for Nanophotonics, Hans-Adolf-Krebs-Weg 1, D-37077 Goettingen, Germany

## Abstract

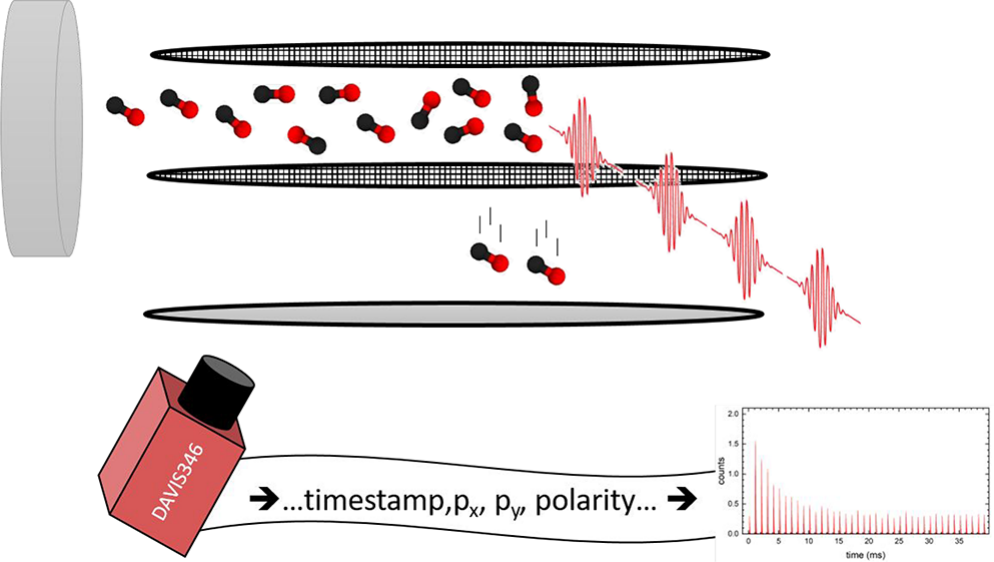

We describe here the application of an inexpensive event-based/neuromorphic camera in an ion imaging experiment operated at 1 kHz detection rate to study real-time velocity-resolved kinetics of thermal desorption. Such measurements involve a single gas pulse to initiate a time-dependent desorption process and a high repetition rate laser, where each pulse of the laser is used to produce an ion image. The sequence of ion images allows the time dependence of the desorption flux to be followed in real time. In previous work where a conventional framing camera was used, the large number of megapixel-sized images required data transfer and storage rates of up to 16 GB/s. This necessitated a large onboard memory that was quickly filled and limited continuous measurement to only a few seconds. Read-out of the memory became the bottleneck to the rate of data acquisition. We show here that since most pixels in each ion image contain no data, the data rate can be dramatically reduced by using an event-based/neuromorphic camera. The data stream is thus reduced to the intensity and location information on the pixels that are lit up by each ion event together with a time-stamp indicating the arrival time of an ion at the detector. This dramatically increases the duty cycle of the method and provides insights for the execution of other high rep-rate ion imaging experiments.

## Introduction

Ion imaging^1^ and its variants—velocity map imaging,^2^ slicing,^3−7^ and 3D imaging^8^—have proven to be powerful tools for the study of chemical dynamics including photodissociation dynamics,^1,9^ crossed-beam scattering,^3,10−12^ and photoelectron spectroscopy.^13,14^ Over the past few years, ion imaging has also been extended to allow the investigation of gas–surface collisions^15−17^ and velocity-resolved surface-reaction kinetics (VRK).^17,18^

In VRK, the reaction is initiated by a pulsed molecular beam and the desorbed reaction products are ionized via nonresonant multiphoton femtosecond-laser ionization 1–2 cm from the surface and detected on an ion imaging detector. The delay between the pulsed molecular beam and the pulsed ionizing laser is scanned under steady-state conditions,^16,17^ averaging the signal to obtain a high quality ion image at each delay. The ion image provides product velocity information that is used to obtain the speed and angular distributions of the products, from which one can determine the product’s flux as well as its flight time from the surface to the laser focus. This provides the necessary information to calculate the product flux vs surface reaction time, referred to as the kinetic trace, a model-independent observable reflecting the speed of the reaction.

Typically, the detection rate of such an experiment is low (10 Hz) and is limited by the acquisition rates of the CCD and CMOS cameras, which record the ion images. For example, to obtain the kinetic trace for CO oxidation on Pd(332) requires about 1 h.^19^ Many catalysts under real reaction conditions change their chemical composition and structure as they become activated or while they are being poisoned through use. It is therefore desirable to develop high-speed methods where transient rates can be quickly measured. Recently, Borodin et al. demonstrated a 1 kHz detection rate using a high-speed CMOS camera capable of acquiring up to 1280 × 800 pixel resolution images at 7.2 kHz.^19^ This camera allowed an ion image to be acquired for each ionizing laser pulse from a 1 kHz Ti:Sa laser. The pulsed molecular beam valve was then operated at 10 Hz starting a new reaction every 100 ms, while ion images were acquired at 1 kHz providing 1 ms time resolution in the kinetic trace. Using this real-time velocity-resolved kinetics approach, the complete kinetic trace of CO desorption and CO oxidation on Pd(332) could be measured within only a few seconds, a dramatic improvement of the data acquisition.

However, these experiments also revealed a severe practical problem typical of imaging experiments performed at high repetition rates. Since the camera needs to record an ion image with Mpixel size for each laser shot, the data acquisition rate can easily reach more than 10 GB/s, limiting the possible duration of any experiment to the time required to fill an on-board high-speed memory, a time that can be as short as a few seconds. The contents of the onboard memory must then be read out before additional ion images can be collected. Thus, the time required for the memory readout to an external solid state storage device becomes the bottleneck to the rate of data acquisition. For example, in the experiments just mentioned, the readout time was several minutes, while the actual acquisition time was only a few seconds.^19^ Consequently, using framing cameras, increasing the frame rate beyond 1 kHz makes no sense. This is unfortunate, as suitable laser operating at 100 kHz (e.g., ytterbium fiber lasers) promise further improvements in data acquisition rates and kinetic time resolution in VRK experiments.

The basic problem of frame-based cameras is the fact that their natural unit of information is a full camera frame with up to 1 Mpixel worth of intensity data. However, a single laser shot in a VRK experiment typically produces only 5–10 ions, lighting up 50–100 pixels of the camera. An ideal read-out system for a high-speed imaging detector would only read out those pixels that actually contain information about these ion detection events. This is accomplished by an event-based or neuromorphic camera,^20−24^ which we show here is ideal for real time VRK experiments.

Event-cameras can serve as detectors for light pulses like those produced by the phosphor screen of an ion imaging detector as they employ a working principle mimicking the human retina^25^ where only changes in brightness are detected. A threshold can be set to reject small brightness changes, suppressing background so that only pixels involved in an ion detection event are detected. In contrast to the high speed framing cameras that record GBytes of data per second, the output of an event-camera-based ion imaging experiment is reduced to a stream of data characterizing only those few pixels whose brightness changes, specifically their spatial coordinates and a time stamp. A recent review summarizes more details and features of commercial event-based sensors.^24^ Recently, event-based sensors with nanosecond time resolution have been applied in velocity map imaging experiments.^26−29^ These new types of cameras offer multimass detection and allow coincidence imaging experiments. However, they suffer from very high costs compared to standard framing cameras.

In this paper, we present the application of a low-cost event-based camera (DAVIS346, IniVation) in a real time VRK experiment recording ion images at a 1 kHz repetition rate. We have chosen the desorption of ammonia (NH_3_), carbon monoxide (CO), and nitric oxide (NO) from Pt(332) as simple systems to test the capabilities of this detection scheme. The time scale of these simple desorption processes can easily be tuned from several milliseconds to >100 ms by changing the surface temperature. This enables an evaluation of the limits of event-based detection. We explore the limit in detection by investigation of the typical time response of the camera to single ion events. The nominal read-out latency of <1 ms of the DAVIS346 provides time stamp accuracy sufficient for these experiments. For all three test cases, the camera is easily able to resolve ions originating from individual laser shots. We estimate that reliable operation is possible up to about 2 kHz allowing real-time velocity resolved experiments with up to 500 μs time resolution. This simple change in the camera technology for the VRK experiment allows reaching the high data acquisition rates demonstrated in ref (19), while avoiding the massive data volume of that work.

## Experimental Set-Up

The apparatus used in this work is similar to that used in earlier VRK experiments.^15−19,30^ It consists of a source chamber equipped with both a Parker Series 9 General Valve and an Even-Lavie type nozzle, producing two pulsed molecular beams. Both beams are skimmed and pass two stages of differential pumping before entering an ultrahigh vacuum chamber with a base pressure of 6 × 10^–10^ mbar where a Pt(332) sample is held. The beams cross at the surface position impinging at an incidence angle relative to the surface normal of 18°. For desorption experiments shown here, we only use the general valve molecular beam with a typical pulse duration of 250 μs. Depending on the desorption rates studied, the nozzle repetition rate can be varied from 0.5 to 50 Hz. The UHV chamber also houses equipment needed for preparing an atomically clean surface. The single crystal Pt(332) sample is mounted to a 4-axis manipulator. Prior to the experiment, the surface is cleaned by Ar^+^ bombardment and heated to 1050 K in 5 × 10^–7^ mbar oxygen for 20 min followed by annealing to 1100 K in UHV for 5 min. The surface quality and cleanliness is checked by low energy electron diffraction (LEED) and Auger electron spectroscopy (AES).

For surface kinetics experiments, the clean and reconstructed sample is then transported in front of the molecular beam behind an ion imaging detector similar to those described previously.^15−19,30^ Molecules leaving the surface are ionized at a distance of about 10 mm from the surface via nonresonant multiphoton ionization using 800 nm pulses from a Ti:Sa laser (120 fs, 1 kHz, 450 μJ per pulse) focused with a 150 mm plano-convex lens. The ions are generated between a repeller and an extractor grid, both with a diameter of 41 mm and separated from one another by 5 mm. A 3 kV pulse applied to the repeller grid extracts the ions, which then pass through a 320 mm long time-of-flight (TOF) tube arriving at a 56 mm diameter Chevron MCP detector. An Einzel lens is located about 19 mm downstream from the repeller which allows switching between spatial ion imaging and velocity map imaging (VMI). The voltage applied to the font face of the MCP detector is time-gated (gate duration 150 ns) to pass ions of a specific mass-to-charge ratio. These ions strike an MCP, producing a pulse of secondary electrons that is accelerated onto a P47 phosphor screen (peak emission 400 nm, 100 ns decay time). A *f*/0.95, 50 mm fixed focal length lens images the light from the phosphor screen onto the event camera (DAVIS346, IniVation, 246 × 320 pixel).

The DAVIS346 event-camera is based on IniVation’s Dynamic Vision Platform and employs an asynchronous read-out.^21^ The output of the camera is a stream of pixel “hits” where each hit contains the pixel coordinates, time stamp in microseconds, and polarity, i.e., brightness increased (ON) or brightness decreased (OFF). Consequently, we only obtain information about those pixels that were actually irradiated by the light flash from the phosphor screen. The pixel coordinates provide spatial information related to ion velocities; this allows discriminating between molecules in the background, in the incident molecular beam, and in those desorbing from the surface. The camera is not actively synchronized to the experiment and runs as a standalone device. However, it offers an input for time stamping of a TTL trigger signal. We use this input to apply a trigger that is synchronized with the molecular beam pulsed valve. This allows us to reference the pixel time stamps to the kinetic time of the experiment. The polarity information is not useful for these experiments; therefore, we only use ON events for our data analysis.

Due to stray light from the pulsed laser, the camera was shielded from laboratory light. Using the camera in the dark leads to an increased noise on the sensor. Hence, we applied a K-noise filter implemented in the camera’s DV software.^31^ The K-noise filter ensures that for each pixel hit in the stream, an ion event is only reported if at least three neighboring pixels are lit up within 500 μs of one another. This simple approach eliminates any dark noise from the stored event-stream.

## Results

The most important feature of the data stream is the time stamp associated with each pixel hit. The time stamp of the DAVIS346 has a nominal temporal resolution of 1 μs; however, the readout electronics lead to loss of temporal resolution, referred to as readout latency, which also depends on several settings of the camera and on the number and intensity of each detected pixel hit. Brewer and Hawks evaluated the optical response of the DAVIS346 and other commercial event-based cameras using a controlled light stimulus.^32^ They also showed that the readout latency determines the temporal resolution—the intrinsic readout latency leads to an uncertainty in the time stamping of each detected pixel hit that can be as much as 1 ms depending on the total number of pixel hits. Since each pulse in the 1 kHz pulse train used in this work determines the time in the kinetic trace, it is sufficient to determine if the time stamp is accurate enough to distinguish ions produced by different laser pulses.

**Figure 1:**
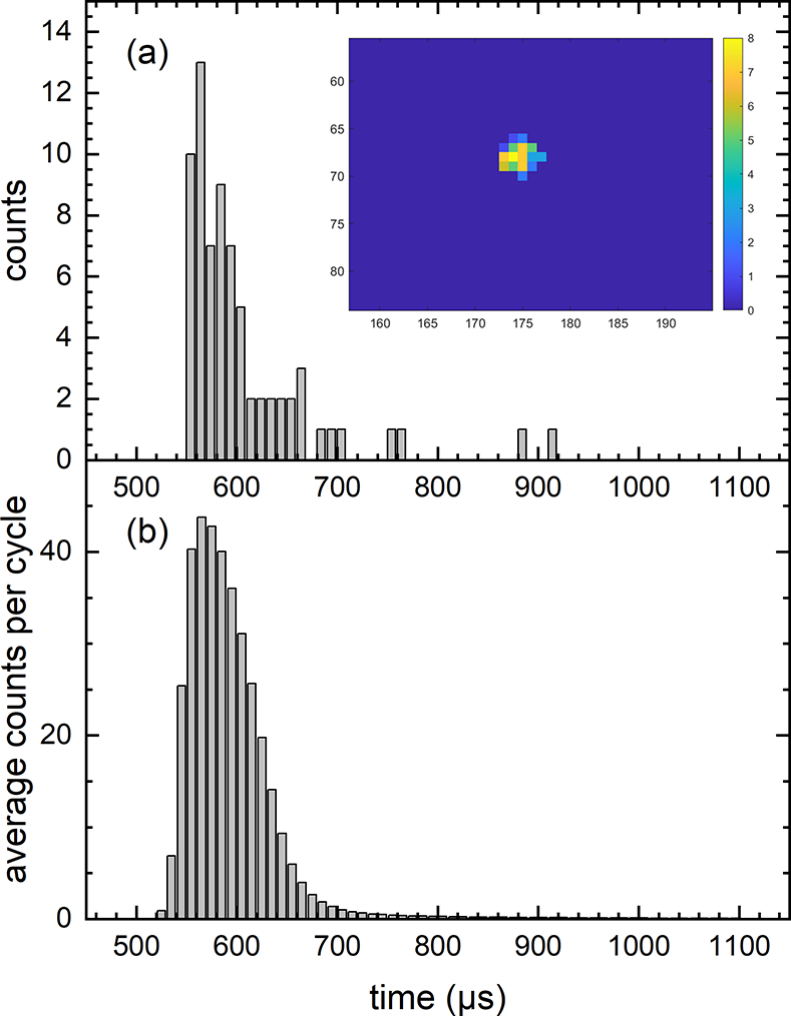
Histogram of the camera time stamps for a range of 500–1000 μs after the detected trigger event using a P47 phosphor. Panel a: distribution of time stamps originating from a single ion detection event. The difference in illuminations allows a single ion event to cause multiple pixel hits resulting in an intensity distribution on the camera. The inset of panel a shows the reconstructed image from the specific pixel hits in the histogram with up to 8 hits per pixel. Panel b: distribution of time stamps from 500 to 1000 μs after nozzle opening averaged over 7533 laser/molecular beam shots. The data show that all pixel hits are time stamped accurately within about 300 μs.

Figure 1a shows a time stamp histogram relative to the external trigger marking the time when the pulsed molecular beam is fired, produced by a single ion detection event in our apparatus. The inset shows the corresponding reconstructed image, reflecting a single ion detection event on the MCP/P47-phosphor detector. The integral of the histogram is proportional to the intensity integral of the image. Hence bright pixels contribute more strongly than dim pixels to the time stamp distribution. Note that the distribution of timestamps is a result of the “time stamping during readout” inside the camera. This leads to the observed time spread in the data (latency), which depends on the readout speed and the event-rate from the detector (scene illumination). Figure 1b shows the time-stamp distribution originating from just the first laser shot after the nozzle opens, averaged over 7533 molecular/laser beam pulses. All pixel hits are time-stamped within ∼300 μs of one another, sufficient to assign each ion detection event to a specific laser pulse of a 1 kHz pulse train.

**Figure 2:**
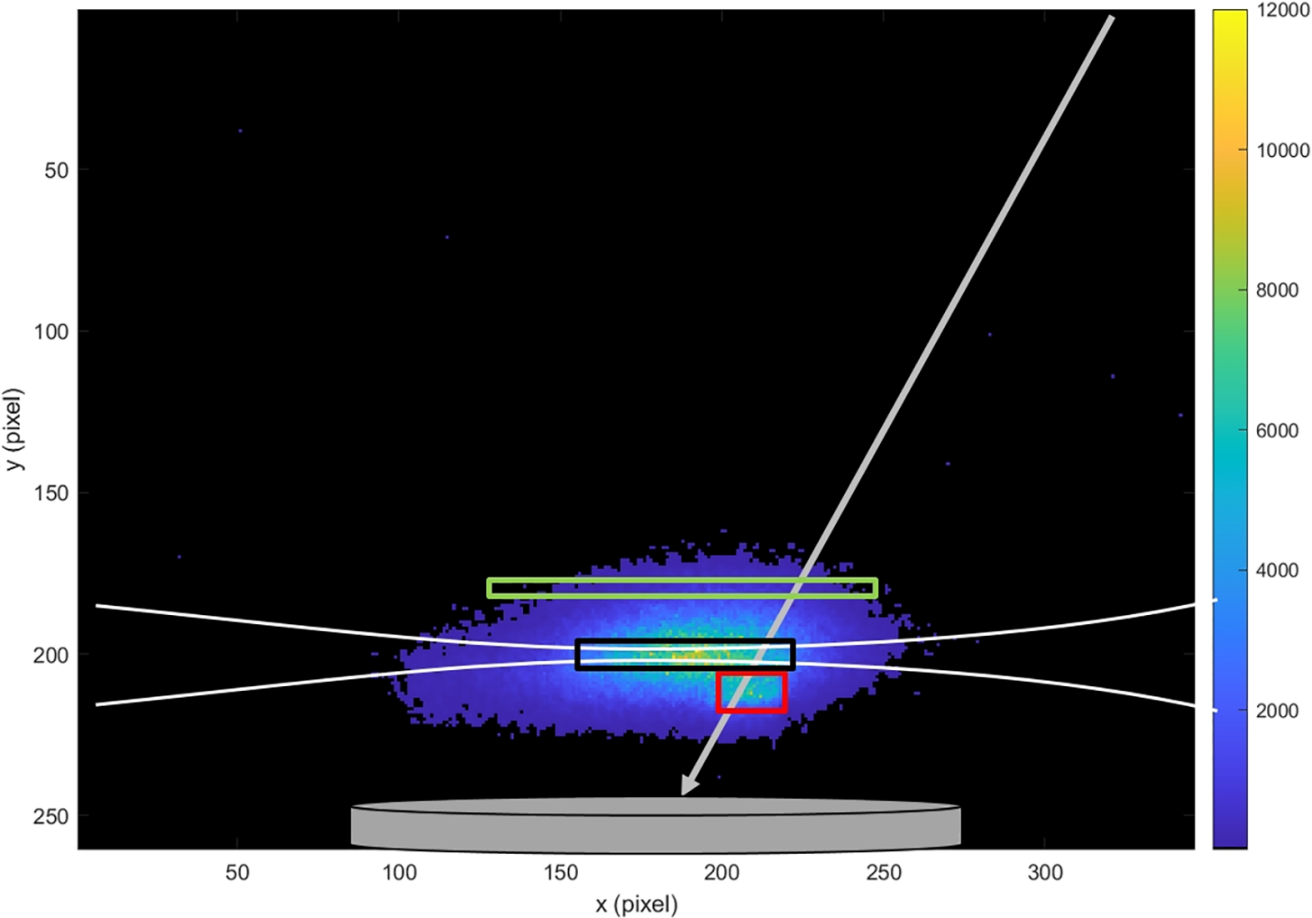
Reconstructed images from the camera hits accumulated over 7598 laser shots for NH_3_ desorption from Pt(332) at 500 K surface temperature. The white lines illustrate the focus shape of the ionizing laser, while the gray line indicates the incident beam. The red square marks the signal originating from ionization of the incident molecular beam. The green rectangle marks the area used to obtain kinetic traces for desorption measurements. The region is selected in a way that is reduced the influence of background signal. The black rectangle shows an area where the NH_3_ desorption signal overlaps with background signal of NH_3_ pressure building up inside the UHV chamber.

Figure 2 shows results for NH_3_ desorption from Pt(332). Specifically, we show an averaged spatial distribution of the ions. The ion image is reconstructed by summing all pixel hits for 7500 molecular beam pulses, where each molecular beam pulse is associated with many laser shots at different times in the kinetic trace that produced ions. The width of the distribution in the vertical direction reflects the velocity spread of the neutral molecules in the experiment. The horizontal spread is determined by the convolution of the ionization volume produced by the focused laser beam (white lines), the size of the pulsed molecular beam incident at the surface and the angular distribution of the desorbing molecules. The black rectangle indicates a region of the ion image where NH_3_ background is strongest. The red rectangle shows the velocities of the incident molecular beam which is moving toward the Pt surface. The green rectangle marks the area used to most sensitively and selectively detect thermally desorbing NH_3_ molecules, which move away from the surface. We can now extract time traces for these different velocity groups.

**Figure 3:**
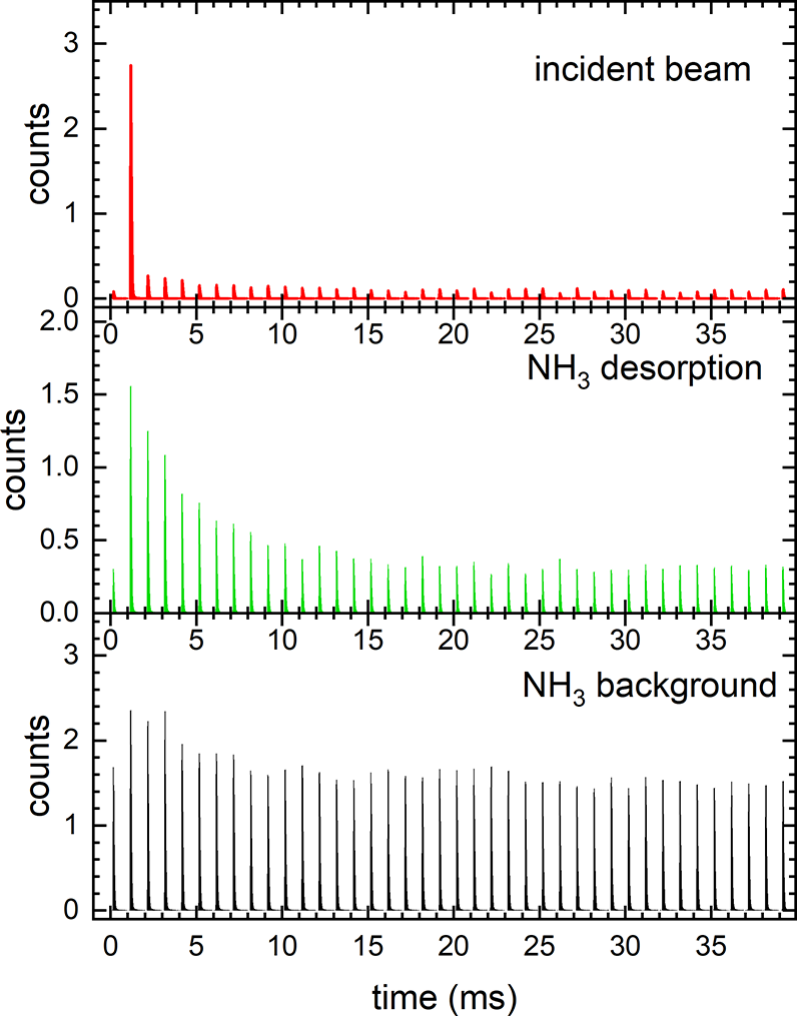
Time traces for the incident molecular beam (red), NH_3_ desorption from Pt(332) (green) and NH_3_ background. The traces result from a histogram of events occurring in the red, green, and black rectangles of Figure 2.

Figure 3 shows histograms of the time stamps for the red, green and black region of interest marked in Figure 2. The red trace in the right panel of Figure 3 shows the average time response of the incident molecular beam with a single sharp peak 1.2 ms after nozzle opening. The green trace in Figure 3 shows the time response of the NH_3_ desorption signal with a rise in intensity at 1.2 ms followed by a decay. Above 20 ms, the signal stays at a nonzero level due to overlapping NH_3_ background in the UHV chamber building up during nozzle operation. The black trace shows an almost constant signal due to a steady NH_3_ background building up inside the UHV chamber which is independent of the delay between ionizing laser and pulsed molecular beam.

The data in Figure 3 demonstrates that the signal originating from individual laser shots can be distinguished on the event-camera. Hence, we may sum up all pixel hits associated with each laser shot to obtain the kinetic trace for desorption.

**Figure 4:**
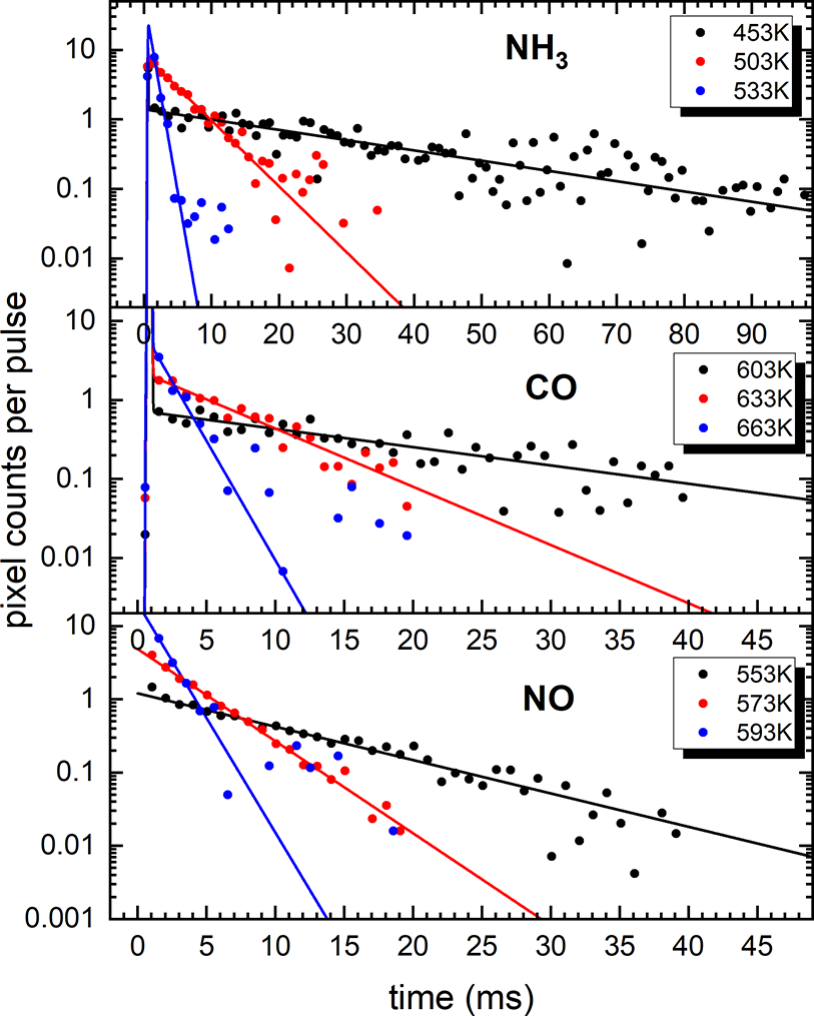
Examples for observed kinetic traces for NH_3_ (top), CO (middle), and NO (bottom) desorption from Pt(332) at three different surface temperatures, respectively. The data show the average pixel hits per molecular beam pulse as a function of time after the nozzle opening. The dots show the raw data points, and the solid lines show the fits to the data assuming first order desorption kinetics.

**Figure 5:**
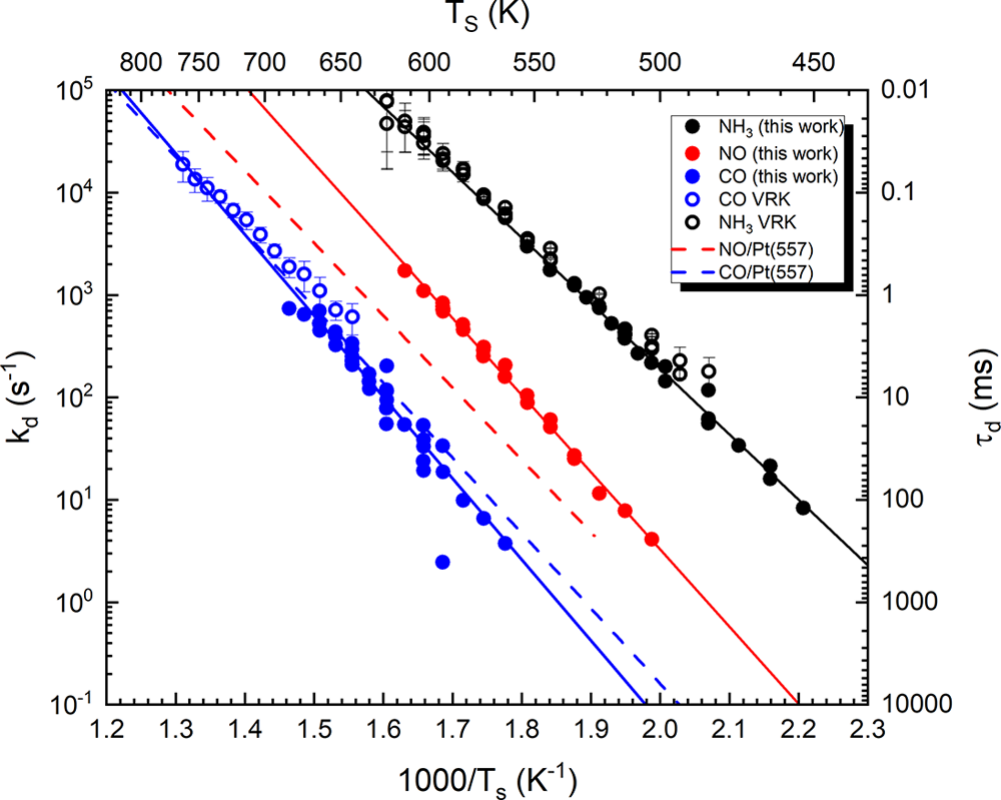
Arrhenius of the measured first-order desorption rate constants as a function of inverse surface temperature for NH_3_ (black), NO (red), and CO (blue) desorption from Pt(332). Solid points show rate constants obtained in this work. The open symbol shows VRK data of ref (33) (CO, open blue) and ref (34) (NH_3_, open black). The dashed lines show Arrhenius curves calculated from MBRS experiments of Lin and Somorjai for Pt(557).^35^

Figure 4 shows examples for kinetic traces obtained in this way for NH_3_, CO, and NO desorption from Pt(332) at several surface temperatures. The closed circles show the raw data, while the lines are fits assuming first order desorption kinetics. We use this to obtain first order rate constants for CO (blue), NO (red), and NH_3_ (black) desorption from Pt(332) which are shown on an Arrhenius plot in Figure 5 in comparison to previous results obtained using conventional VRK^33,34^ (open symbols) and molecular beam relaxation spectroscopy (MBRS, dashed lines).^35^ The solid lines are Arrhenius fits to the temperature dependent rate constants. Table 1 shows the resulting activation energies and prefactors.

**Table 1:** Arrhenius Activation Energies and Prefactors Obtained from Figure 5^a^

	activation energy/eV	prefactor/s^–1^
CO/Pt(332)	1.57 ± 0.11	4.98 × 10^14^
NO/Pt(332)	1.50 ± 0.04	3.90 × 10^15^
NH_3_/Pt(332)	1.27 ± 0.06	1.19 × 10^15^

a Errors represent 95% confidence interval.

## Discussion

The time stamp histograms in Figure 1 demonstrate that the event-based camera is able to resolve ion hits from individual laser pulses at 1 kHz. While the P47 phosphor screen causes a sudden excitation of the sensor, the camera time stamp shows a latency of less than 300 μs. It seems therefore possible to use the camera for experiments running at up to 2 kHz repetition rate with a kinetic time resolution of 500 μs. It is worth noting that the latency might increase for high count-rates, which are however unlikely in ion imaging applications where a single laser pulse typically produces 2–10 ions with each ion exciting 4 × 4 pixels. Consequently, even at 2 kHz detection rate only 3.2 × 10^5^ pixel per second would be excited. This is still significantly lower than the maximum throughput of (8–12) × 10^6^ pixel hits per second of the DAVIS346 camera. We want to bring to the reader’s attention that it is possible to increase the spatial resolution of the camera to a subpixel level by using centroiding algorithms that have been developed in the past.^36−39^ The processing can even be more efficient since the step of identifying bright pixels of a full frame image is unnecessary. In our experiments, an increased resolution has no benefit since we detect broad thermal velocity distributions. The only advantage might be an increased signal-to-noise ratio since all ions contribute only a single count to the signal.

The most significant advantage over using standard high-speed framing cameras is the aspect of data storage. For the example shown in Figure 2 and Figure 3, we accumulated a signal of 187500 laser shots (7500 molecular beam pulses at 25 Hz). A typical high-speed camera with the same number of pixels and 12bit resolution would require storage of about 22GB of image data, which would need to be stored to an external hard drive. In contrast, the primary AE4DAT file stored by the DAVIS346 camera software is only 70 MB in size. In addition, an event-camera with a higher pixel number for increased resolution, will not significantly increase the data size since only active pixels are stored. Furthermore, the DAVIS346 camera stores the data directly on the hard drive of the computer while high-speed framing camera typically first store the images in an internal RAM and transfer them to the computer after the measurement is finished. The readout process can easily take several minutes and leads to a decrease in the duty cycle of the experiment.

The measured desorption rates cover a significant range from 5 to 1000 s^–1^ which corresponds to residence times of the molecules from 1 to 200 ms. While a desorption rate of *k*_d_ = 1000 s^–1^ represents the upper limit for the time resolution of a 1 kHz repetition rate laser, the data shows that the high detection rate is also useful to measure slow kinetic rates as suggested by Borodin et al.^19^ All velocity resolved kinetics suffer from the problem that they probe the flux of molecules leaving the surface, which decreases with decreasing desorption rate. In conventional velocity-resolved kinetics experiments this results in a rapidly increasing measurement time. Working at high acquisition rates and high duty cycle can partially overcome this problem since it increases the sampling rate by several orders of magnitude. Hence, a decreasing desorption flux is compensated by a high number of sample points. Consequently, high repetition rate detection allows an extension of kinetic data to lower surface temperatures which increases the accuracy of extracted Arrhenius activation energies.

Finally, the comparison of Figure 5 shows that the desorption rate constants obtained in this work (solid circles) agree very well to previous VRK experiments^33,34^ (open circles) for CO and NH_3_. The VRK data usually extend to higher surface temperatures than the 1 kHz measurements due to the use of a shorter molecular beam pulse and the smaller time increments available in a delay scan approach. However, the 1 kHz detection data of this work extend to lower surface temperatures which are typically not covered in VRK experiments because of the very low flux from the surface and the resulting extensive measurement times. The high sampling rate of this work makes these temperatures accessible. The dashed red lines in Figure 5 show Arrhenius curves calculated from the parameters obtained by Lin and Somorjai^35^ for CO (blue) and NO (red) desorption from Pt(557) using MBRS. Pt(557) has the sasme step density of 16.6% as Pt(332) but a different step geometry. The comparison shows that the parameters of Lin and Somorjai agree very well with the rate constants obtained for CO desorption from Pt(332) of this work. In contrast, the MRBS results for NO desorption from Pt(557) are significantly different from those obtained for desorption from Pt(332) in this work. This might be an indication for the influence of different step geometries on the binding energy of NO.

## Conclusions

We have shown in this paper the possibility to apply a low-cost event-based camera to measure real-time kinetics at a sampling rate of 1 kHz. In contrast to previous experiments using high-speed framing cameras, this approach has several advantages. First, the camera has a much more compact design and lower power consumption. Second, the event-based camera in this work costs 10% as much as a corresponding framing camera system. Third, the data volume produced by a real-time VRK experiment is dramatically reduced. High-speed framing cameras exhibit data rates of several GB/s making long-term measurements impossible. In contrast, an event-based camera only produces several kB of data per second and even a 20 min continuous measurement uses only a few 100 MB of storage capacity. However, low-cost event-cameras have issues with accurate time stamping of pixel hits. It is therefore necessary to work under conditions where the pixel hits from individual laser shots can be clearly distinguished from each other.

We emphasize the importance of a fast phosphor since event-based cameras are sensitive to photon flux and not to the total number of emitted photons like integrating CMOS/CCD framing cameras. We have also performed experiments using a common P43 phosphor screen, which has a decay time of 1.6 ms. Besides a significantly reduced count rate on the camera, it was not possibly to separate ions from single laser shots in time. For the DAVIS346 camera used in this work in combination with a fast phosphor screen, we estimate the maximum possible ionization rate to be around 2 kHz, which would enable measurements with a continuous time resolution of 500 μs, which is sufficient for a variety of applications. We emphasize that many imaging experiments operated at high repetition rates do not require an event camera with ns-time stamp accuracy if they only detect single *m*/*z* ratios. The DAVIS346 is about 5% the cost of such event cameras that provide nanosecond time resolution. We hope that this work might make the use of event cameras more available in ion imaging applications.
